# Involvement of langerin in the protective function of a keratan sulfate-based disaccharide in an emphysema mouse model

**DOI:** 10.1016/j.jbc.2023.105052

**Published:** 2023-07-15

**Authors:** Yuki Ohkawa, Noriko Kanto, Miyako Nakano, Reiko Fujinawa, Yasuhiko Kizuka, Emma Lee Johnson, Yoichiro Harada, Jun-ichi Tamura, Naoyuki Taniguchi

**Affiliations:** 1 Department of Glyco-Oncology and Medical Biochemistry, Osaka International Cancer Institute, Osaka, Japan; 2 Graduate School of Integrated Sciences for Life, Hiroshima University, Higashi-Hiroshima, Hiroshima, Japan; 3 Glycometabolic Biochemistry Laboratory, RIKEN Cluster for Pioneering Research, Wako, Saitama, Japan; 4 Institute for Glyco-core Research (iGCORE), Gifu University, Gifu, Japan; 5 Department of Molecular Biochemistry and Clinical Investigation, Graduate School of Medicine, Osaka University, Osaka, Japan; 6 Department of Life and Environmental Agricultural Sciences, Faculty of Agriculture, Tottori University, Tottori, Japan

**Keywords:** glycobiology, carbohydrate function, chronic obstructive pulmonary disease (COPD), dendritic cell, inflammation, CapG, langerin, keratan sulfate

## Abstract

Chronic obstructive pulmonary disease (COPD), which includes emphysema and chronic bronchitis, is now the third cause of death worldwide, and COVID-19 infection has been reported as an exacerbation factor of them. In this study, we report that the intratracheal administration of the keratan sulfate-based disaccharide L4 mitigates the symptoms of elastase-induced emphysema in a mouse model. To know the molecular mechanisms, we performed a functional analysis of a C-type lectin receptor, langerin, a molecule that binds L4. Using mouse BMDCs (bone marrow-derived dendritic cells) as langerin-expressing cells, we observed the downregulation of *IL-6* and *TNFa* and the upregulation of *IL-10* after incubation with L4. We also identified CapG (a macrophage-capping protein) as a possible molecule that binds langerin by immunoprecipitation combined with a mass spectrometry analysis. We identified a portion of the CapG that was localized in the nucleus and binds to the promoter region of *IL-6* and the *TNFa* gene in BMDCs, suggesting that CapG suppresses the gene expression of *IL-6* and *TNFa* as an inhibitory transcriptional factor. To examine the effects of L4 *in vivo*, we also generated *langerin*-knockout mice by means of genome editing technology. In an emphysema mouse model, the administration of L4 did not mitigate the symptoms of emphysema as well as the inflammatory state of the lung in the *langerin*-knockout mice. These data suggest that the anti-inflammatory effect of L4 through the langerin-CapG axis represents a potential therapeutic target for the treatment of emphysema and COPD.

Chronic obstructive pulmonary disease (COPD), which includes emphysema and chronic bronchitis, is the third most common cause of death in the world. The development of chronic bronchitis in the lung induced by long-term exposure to tobacco leads to bronchial obstruction and respiratory failure, conditions that are not fully reversible and which eventually cause emphysema and COPD in humans (1). Tobacco smoke can activate a variety of immune cells including macrophages, neutrophils, T-lymphocytes, B-lymphocytes, and dendritic cells that are present in the lung (2). The activated immune cells secrete various inflammatory mediators including chemokines, cytokines, growth factors, and proteolytic enzymes, and these in turn, stimulate each other cells (3, 4). These inflammatory mediators exacerbate bronchitis and induce bronchial obstruction. In such cases, corticosteroids and related, anti-inflammatory drugs are administrated to the patients to suppress the progression of COPD (5). Concerning the suppression of the inflammatory state, dendritic cells would be potential targets because they play a central role in the initiation and acceleration of immune responses (6). By sensing the harmful substances in tobacco smoke, dendritic cells activate the immune cell network to protect the body from them, but its responses often damage the bronchi, which leads to the onset and progression of emphysema and COPD (7). The infections such as COVID-19 can also exacerbate the symptoms by activating immune cells (8).

Langerin, a C-type lectin receptor that is expressed in Langerhans cells, is a type II membrane protein that contains a C-terminal carbohydrate recognition domain in its extracellular region (8). Langerhans cells are a subtype of dendritic cells that are located in the epidermis and it has been recently shown that a part of the resident dendritic cells in other organs also expresses langerin (9, 10). Importantly, it was demonstrated that langerin functions as a natural barrier against the HIV-1 (human immunodeficiency virus 1), the influenza virus, and related viruses by recognizing the glycans that are attached to their envelope proteins (11, 12, 13, 14). After being captured by langerin, they are incorporated by endocytosis, then degraded by lysosomes, and the antigen presentation then proceeds. It is possible that langerin as well as other lectin receptors could capture other pathogens by recognition of glycans, but to verify this a more detailed structural analysis would be needed to permit their potential glycan ligands to be identified.

Tateno *et al*. identified some potential glycan structures that are recognized by langerin (15). Using a langerin-Fc chimera and a glycan array, they found that langerin-Fc binds strongly to the 6′-sulfo-LacNAc (galactose-6-sulfate linked by a β1,4 linkage to *N*-acetylglucosamine). Importantly, in *in vitro* assays, we also demonstrated that 6′-sulfo- and 6-sulfo-LacNAc (galactose-6-sulfate β1,4-linked to *N*-acetylglucosamine-6-sulfate) binds to langerin more strongly than 6′-sulfo-LacNAc (16). These disaccharides are components of the glycosaminoglycan of the keratan sulfate proteoglycan (Fig. S1), therefore, these results indicate that the keratan sulfate side chain would be a natural ligand for langerin.

We refer to these, 6′-sulfo- and 6-sulfo-LacNAc disaccharides, as L4, and, in an independent study, our research group previously reported on the potential function of L4 against an emphysema mouse model. It was demonstrated that the intratracheal administration of L4 modulated the inflammatory state in the lung and mitigated the pathology of emphysema (17). While these data suggest therapeutic potentials of L4 against emphysema, we had no knowledge regarding the target of L4 and no information concerning the mechanism responsible for the action of L4. Taking these background findings into consideration we report on our attempts to address these questions in this study by using langerin-expressing cells and an emphysema mouse model in *langerin*-knockout mice. Our findings indicate that the protective function of L4 against emphysema represents one of the mechanisms in in this process.

## Results

### L4 suppresses the gene expression of inflammatory cytokines in BMDCs

**Figure 1:**
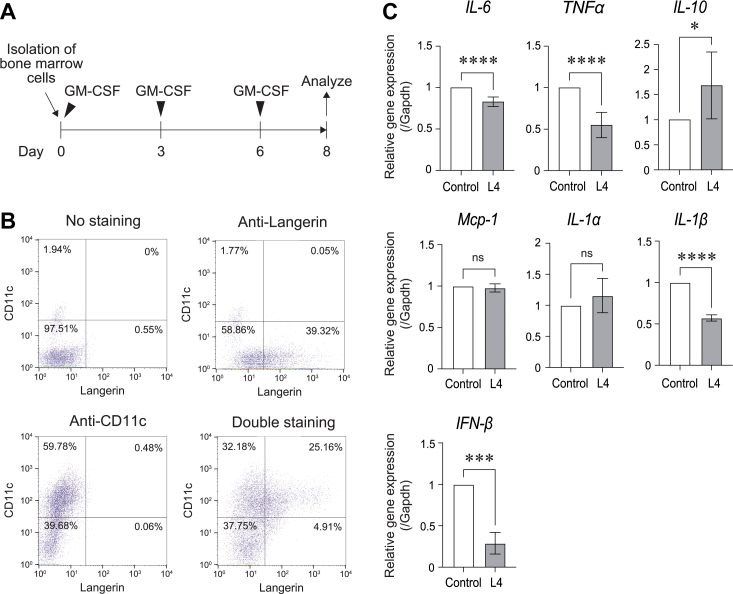
**L4 downregulated inflammatory cytokines in BMDCs**. *A*, a time course for the isolation and differentiation of mouse bone-marrow cells into BMDCs (bone-marrow-derived dendritic cells). *B*, the expression of langerin and CD11c in BMDCs was examined by flow cytometry. The X-axis indicates langerin expression and the Y-axis indicates CD11c expression. The numbers indicate the percentages of the cells existing each square area. *C*, the gene expression of IL-6, TNFα, IL-10, Mcp-1, IL-1α, IL-1β, and IFN-β in BMDCs after incubation with L4 (100 μg/ml) for 2 days examined by qRT-PCR analysis. The expression levels after incubation with L4 were normalized to the levels without L4. The expression levels indicated as mean ± SD of three independent experiments. ∗*p* < 0.05, ∗∗∗*p* < 0.001, ∗∗∗∗*p* < 0.0001, ns: not significant.

It is known that the expression of langerin is restricted in dendritic cells. To examine the function of langerin, we first analyzed the expression of langerin in a well-established mouse follicular dendritic cell line, FL-YB mice. However, no langerin expression was observed in that cell line. For this, we next isolated murine bone marrow cells and differentiated them into dendritic cells by incubation with granulocyte-macrophage colony-stimulating factor (GM-CSF) (Fig. 1*A*) (18, 19). These bone marrow-derived dendritic cells (BMDCs) expressed langerin as well as CD11c, a dendritic cell marker, in 39.32 % and 59.78 % of the total cells, respectively (Fig. 1*B*) and 25.16 % of the total cells were double positive (Fig. 1*B*). Using this information, we incubated the cells with L4 for 2 days and then, analyzed gene expression by quantitative RT-PCR. As shown in Figure 1*C*, L4 downregulated the inflammatory cytokines *IL-6*, *TNFα*, *IL-1β*, and *IFN-β*, while upregulated an anti-inflammatory cytokine *IL-10*. The expression of *Mcp-1* and *IL-1α* did not change. These data suggest that L4 suppressed the inflammatory status of langerin-expressing BMDCs.

### CapG (macrophage-capping protein) is immunoprecipitated with langerin in BMDCs

**Figure 2:**
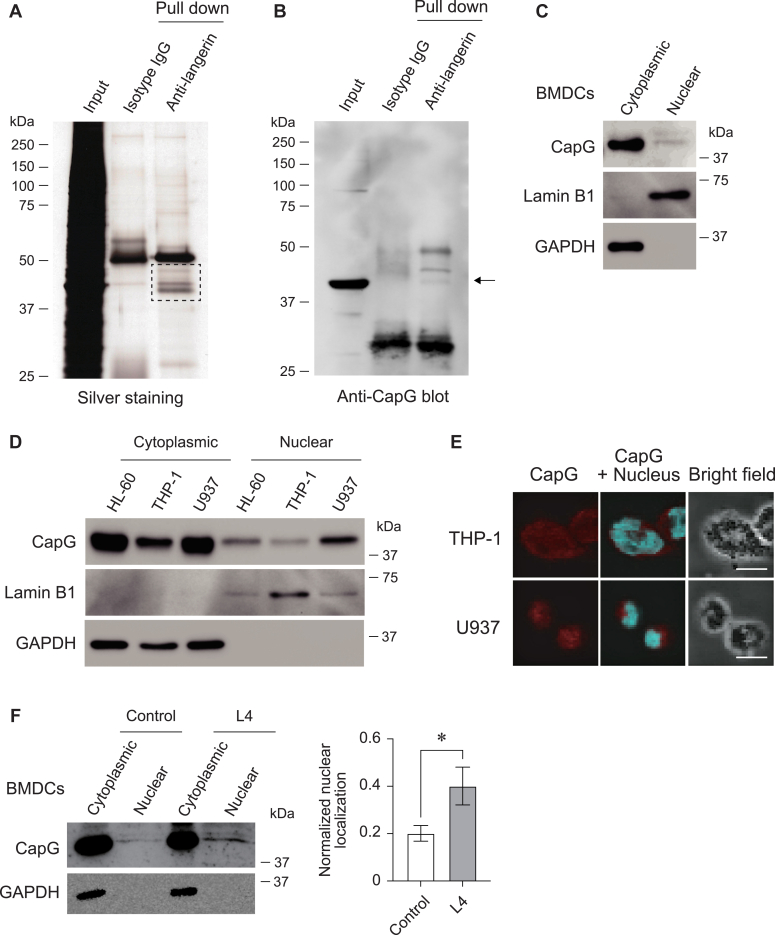
**CapG was identified as a langerin-binding molecule and localized in the nucleus.***A*, silver staining of the SDS-PAGE gel. The immunoprecipitates prepared from BMDCs with an anti-langerin antibody and a non-relevant IgG separated in SDS-PAGE were then subjected to silver staining. The dashed square area was cut out and the contents analyzed by mass spectrometry. *B*, Western blotting of the immunoprecipitates prepared from BMDCs with an anti-langerin antibody and a non-relevant IgG. The membrane was blotted with an anti-CapG antibody. *Arrow* indicates CapG. *C*, subcellular localization of CapG in BMDCs examined by western blotting. Nuclear-rich fraction and cytoplasm-rich fraction were prepared and analyzed. Lamin B1 was used as a maker for nuclear fraction and GAPDH was used as a marker for cytoplasmic fraction. *D*, subcellular localization of CapG in HL-60, THP-1, and U937 cells examined by western blotting. *E*, immunocytochemical staining of THP-1 and U937 cells with an anti-CapG antibody and DAPI. Red indicates CapG and blue indicates nuclear. Bars indicate 10 μm. Images were pictured under confocal microscopy. *F*, subcellular localization of CapG in BMDCs after incubation with L4 (100 μg/ml) for 2 days examined by western blotting. GAPDH was used as a marker for the cytoplasmic fraction. Independent experiments were performed and the normalized nuclear localization of CapG after incubation with or without L4 is shown in the right panel. ∗*p* < 0.05.

The molecules that functioned with langerin in a cooperative manner have not been identified yet. For this, we next attempted to identify the molecules that bind with langerin. In BMDCs, the cell lysate was immunoprecipitated with an anti-langerin antibody and SDS-PAGE was performed with the resulting immunoprecipitates. As shown in Figure 2*A*, we found unique bands in the molecular weight range of 37 to 50 kDa after visualizing the proteins by silver staining in molecules that were immunoprecipitated with an anti-langerin antibody. To identify these proteins, we cut the bands out and analyzed them by mass spectrometry. A total of 11 proteins were identified as langerin-binding molecules and are listed in Table 1. Among them, we confirmed the binding between langerin and CapG (a macrophage-capping protein) in the immunoprecipitate with an anti-CapG antibody by western blotting (Fig. 2*B*). In addition, we also analyzed the binding between them in HEK293 cells. We overexpressed mouse langerin and mouse CapG together and immunoprecipitation with an anti-langerin antibody was then performed. The immunoprecipitate was blotted with an anti-CapG antibody. As shown in Fig. S2, CapG was indeed detected as a molecule that binds to langerin. Moreover, we also found that langerin and CapG are endogenously expressed in a human monocytic leukemia cell line, THP-1 cells. In THP-1 cells, the binding between langerin and CapG and the association between them in the plasma membrane was also confirmed (Fig. S3). These data indicate that CapG would function with langerin cooperatively in BMDCs.

**Table 1:** Identified molecules as langerin-associated proteins

Name	Gene Name	Identified peptide sequence	Coverage (%)
Langerin	Cd207	SQILSLETSMK	16
		SDAQMLK	
		RPYVQTTE	
		KAHLTSVSSESEQK	
		AHLTSVSSESEQK	
		VQGLQNSLENVNK	
IgG2a secreted form	N/A	VNNRALPSPIEK	6
		VDKKIEPR	
		ALPSPIEK	
60S ribosomal protein L4	Rpl4	MMNTDLSR	2
Zinc finger protein 609	Anf609	CKKPSSLK	1
Transmembrane protease serine 13	Tmprss13	NKPGVYTK	1
WD repeat-containing protein 81	Wdr81	EAGLLAAVTLTQK	1
Macrophage-capping protein	Capg	VSDATGQMNLTK	3
UPF0711 protein C18orf21 homolog	N/A	SNAATAANKASPK	6
FK506-binding protein-like	Fkbpl	VLAVDPKNR	3
RCC1 and BTB domain-containing protein 2	Rcbtb2	REFDNPDTADLK	2
Renalase	Rnls	QREQLK	2

### L4 enhanced nuclear localization of CapG in BMDCs

Human and mouse CapG harbor a putative nuclear localization signal and their amino acid sequence lack the nuclear export signal (20). To further examine the function of CapG, we analyzed the subcellular localization of CapG in murine BMDCs and in a human promyelocytic leukemia cell line: HL-60, a human monocytic leukemia cell line: THP-1, and in U937 cells: a human diffuse histiocytic lymphoma cell line. We prepared cytoplasmic and nuclear fractions from these cells and performed western blotting. As shown in Figure 2, *C* and *D*, a portion of the CapG was detected in the nuclear fractions. The larger amount of GAPDH (glyceraldehyde-3-phosphate dehydrogenase), however, was detected in cytoplasmic fractions and Lamin B1 was enriched in nuclear fractions in these samples. We also stained the cells with an anti-CapG antibody and DAPI in THP-1 and U937 cells. As shown in Figure 2*E*, CapG was clearly localized in the nucleus in these cells. In addition, to determine if the CapG localization is regulated by an L4 treatment, we again analyzed the subcellular localization of CapG in BMDCs after incubation with L4. Interestingly, as shown in Figure 2*F*, the amount of CapG present in the nuclear fraction was increased by the L4 treatment. These data indicate that L4 promoted the nuclear localization of CapG in langerin-expressing BMDCs.

### CapG functions as a transcription factor to suppress TNFα gene expression in BMDCs

**Figure 3:**
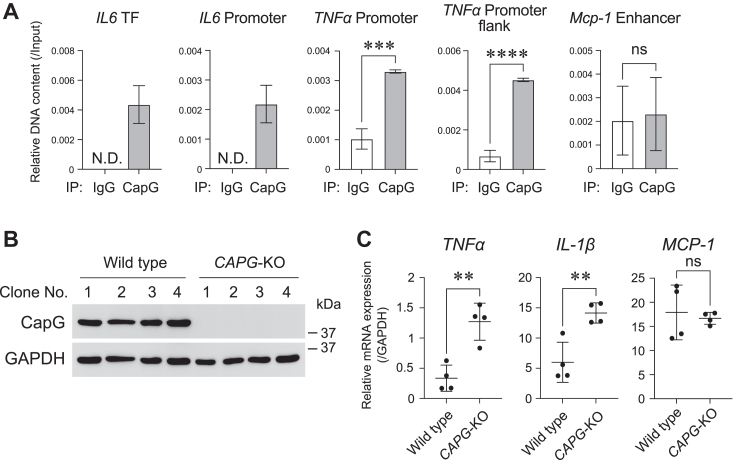
**CapG functioned as an inhibitory transcription factor to suppress TNFa gene expression**. *A*, ChIP-qPCR analysis targeting the promoter or enhancer region for the *IL-6*, *TNFα*, and *Mcp-1* genes. Genomic DNA of BMDCs was fragmentated and immunoprecipitated with an anti-CapG antibody or a non-relevant IgG, then, immunoprecipitated DNAs were purified and analyzed by qPCR analysis. Relative DNA content relative to input samples were indicated as the mean±SD. N.D.: not detected. ∗∗∗*p* < 0.001, ∗∗∗∗*p* < 0.0001, ns: not significant. TF: transcription factor binding site. *B*, Western blot analysis of CapG in clonal cell lines derived from THP-1 cells. After transfection of a vector to knockout *CAPG* gene by genome editing in THP-1 cells, four clonal cell lines were established and analyzed. GAPDH was used as a loading control. *C*, the gene expression level of *TNFα*, *IL-1β*, and *MCP-1* in *CAPG* knockout cells (*CAPG*-KO) and control cells were examined by qRT-PCR analysis. Before the analysis, the cells were differentiated by incubation with PMA (phorbol 12-myristate 13-acetate) (20 nM) for 2 days. The expression levels were normalized to GAPDH genes. ∗∗*p* < 0.01, ns: not significant.

Concerning the nuclear localization of CapG, we hypothesized that CapG was involved in the regulation of gene expression. To address this, we performed ChIP (chromatin immunoprecipitation) analyses of BMDCs. After fixation of the cells, DNA fragmentation and immunoprecipitation with an anti-CapG antibody were carried out. DNA fragments bound to CapG were purified and pPCR (quantitative polymerase chain reaction) targeting the *IL-6* promoter or transcription binding region, *TNFα* promoter region, and *Mcp-1* enhancer region were also performed (Fig. S4). Interestingly, as shown in Figure 3*A*, qPCR targeting *IL-6* was successful only in the immunoprecipitate prepared with an anti-CapG antibody, while it was not observed in the case of the immunoprecipitate prepared with non-relevant IgG. We also observed that the DNA fragments of the *TNFα* promoter region were enriched in the immunoprecipitate prepared with an anti-CapG antibody, while this enrichment was not observed in the sample prepared with non-relevant IgG. The DNA fragments of the *Mcp-1* enhancer region were not enriched significantly. In addition, to analyze the roles of CapG in the regulation of *TNFα* gene expression, we knocked out the *CAPG* gene in THP-1 cells by the genome editing technology, CRISPR/Cas9. The knocking out of the *CAPG* gene was confirmed by western blotting as shown in Figure 3*B*. Using these cells, we analyzed the expression level of the *TNFα*, *IL-1β*, and *MCP-1* genes by qRT-PCR and the results showed that the *CAPG*-knockout cells expressed the *TNFα* and *IL-1β* genes more strongly than the control cells (Fig. 3*C*). These data suggest that CapG binds to the *TNFα* promoter region and suppresses their gene expression as an inhibitory transcription factor.

### L4 did not suppress the expression of genes that encode for inflammatory cytokines in langerin-knockout BMDCs

**Figure 4:**
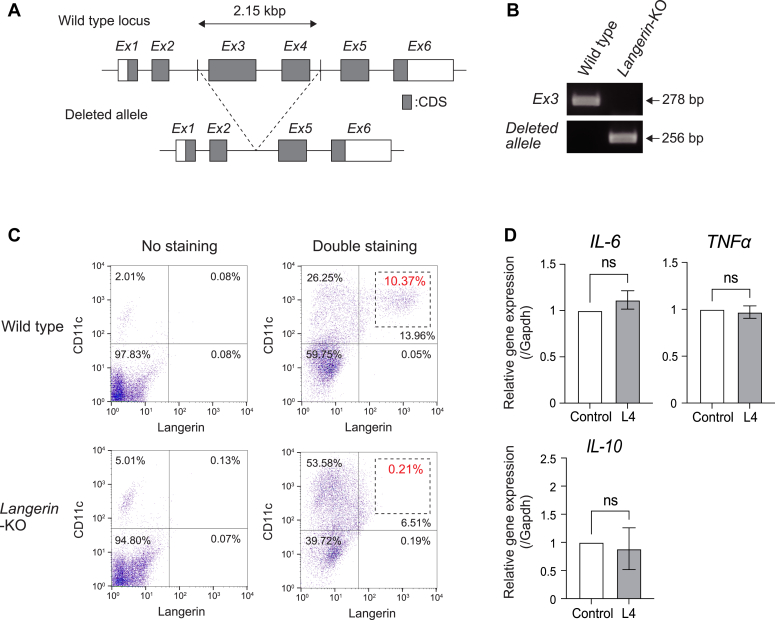
**L4 did not downregulate inflammatory cytokines in BMDCs prepared from langerin-knockout mice.***A*, the locus of the *langerin* gene in wild type mice and *langerin*-knockout mice. The *exon No.3* (*E x 3*) and *exon No.4* (*E x 4*) of *langerin* gene were deleted by genome editing in *langerin*-knockout mice. *B*, PCR analysis targeting *exon No.3* (*E x 3*) of *langerin* gene and deleted allele. Genomic DNAs were prepared from wild type mouse and *langerin*-knockout mouse (*langerin*-KO). *C*, the expression of langerin and CD11c in BMDCs prepared from wild type mice and *langerin*-KO mice examined by flow cytometry. The X-axis indicates langerin expression and Y-axis indicates CD11c expression. The numbers shown by dushed squares indicates the percentage of the langerin-expressing cells. *D*, the gene expression of *IL-6*, *TNFα*, and *IL-10* in BMDCs prepared from *langerin*-KO mice after incubation with L4 (100 μg/ml) for 2 days was examined by qRT-PCR analysis. The expression levels after incubation with L4 were normalized to the levels without L4. The expression levels were indicated as mean ± SD of three independent experiments. ns: not significant.

Our group previously reported that L4 binds to langerin as described above, however, the functional meaning of this event has not yet been demonstrated *in vivo*. For this, we generated a *langerin*-knockout mouse with genome editing technology, CRISPR/Cas9. The *langerin*-knockout mouse lacks exon three and exon four of *langerin* as illustrated in Figure 4*A*. The genomic PCR analysis confirmed exon three of *langerin* had been knocked out and that the generation of the deleted allele occurred in a knockout mouse (Fig. 4*B*). The sequencing analysis of genomic DNA also confirmed the deletion (Fig. S5). In BMDCs prepared from *langerin*-knockout mice, the expression of langerin was not observed by flow cytometric analysis (Fig. 4*C*). Additionally, to determine whether L4 functions through langerin, we examined the gene expression of *IL-6*, *TNFα*, and *IL-10* in BMDCs prepared from the *langerin*-knockout mice. Very interestingly, incubation with L4 did not result in the downregulation of those genes, as evidenced by a quantitative RT-PCR analysis in BMDCs prepared from *langerin*-knockout mice (Fig. 4*D*). These data suggest that the anti-inflammatory function of L4 proceeded via langerin.

### L4 suppressed the bronchial obstruction through langerin in an emphysema mouse model

**Figure 5:**
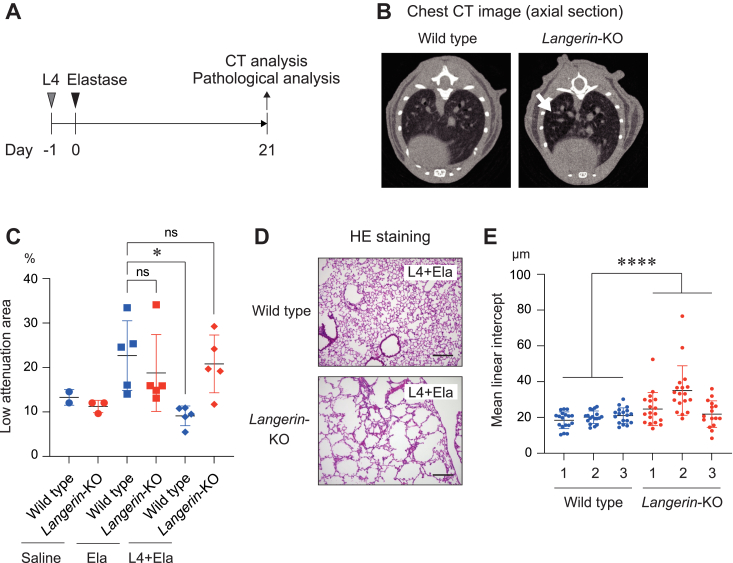
**L4 suppresses bronchial obstruction through langerin in an emphysema mouse model.***A*, the schedule for the administration of L4 (1 mg/mouse) and elastase (4 U/mouse). At 21 days after the administration of elastase, CT analysis, and pathological analyses were performed. *B*, representative images of the CT analysis in the lung of wild type and *langerin*-KO mouse after administration of L4 and elastase. *Arrow* indicates low attenuation of X-ray in the lung of *langerin*-KO mouse. *C*, the percentages of low attenuation area in the lung of wild type and *langerin*-KO mice after administration of elastase with or without L4 examined by CT analysis. Each five mice in each group were analyzed after elastase treatment. ∗*p* < 0.05. ns: not significant. *D*, representative images of HE staining of the lung sections prepared from wild type and *langerin*-KO mice after administration of L4 and elastase. Bars indicate 200 μm. *E*, the mean linear intercept for the lung prepared from wild type (n = 3) and *langerin*-KO mice (n = 3) after administration of L4 and elastase. Random 16−18 lines in each mouse were measured. ∗∗∗∗*p* < 0.0001.

An elastase-induced emphysema mouse model is a well-established animal model in the respiratory research field (21). Elastase is an enzyme that catalyzes the degradation of elastin which is a major component of the extracellular matrix (22). The administration of elastase into the tracheae induces bronchial obstruction and bronchitis which are similar to the symptoms of emphysema in humans. To analyze the function of L4 against emphysema, we administrated L4 and elastase into the tracheae of *langerin*-knockout mice and wild-type mice (Fig. 5*A*). At 21 days after the administration, we assessed the state of emphysema with CT (computed tomography) analysis and found that bronchial obstruction had been induced as indicated by low attenuation of X-rays observed in *langerin*-knockout mice (Fig. 5*B*). To quantify this, we also assessed the LAA (low attenuation area) of the lungs in *langerin*-knockout mice and wild type mice after the administration of elastase with and without L4. The administration of elastase induced an equal level of bronchial obstruction between *langerin*-knockout mice and wild type mice because the LAA was similar between these two groups (Fig. 5*C*, central two groups). On the other hand, and very interestingly, the administration of L4 before the elastase treatment significantly decreased LAA in wild type mice, but not in the *langerin*-knockout mice (Fig. 5*C*, right two groups). Moreover, we performed pathological analyses in these mice and observed severe bronchial obstructions in the *langerin*-knockout mice, not in the wild0type mice after the administration of L4 and elastase (Fig. 5*D*). The mean linear intercept of the lung was increased in *langerin*-knockout mice compared to wild-type mice (Fig. 5*E*). Additionally, pulmonary fibrosis is sometimes combined with emphysema, which induces more severe respiratory failure (23). To analyze the extent of fibrosis, we performed Masson’s trichrome staining. As shown in Fig. S6, lung sections of the *langerin*-knockout mouse showed evidence of advanced fibrosis, but this was not observed in the wild type mouse. These data strongly suggest that L4 has a protective function against emphysema and that it is exhibited through langerin.

### L4 mitigated the inflammatory state through langerin in the emphysema mouse model

**Figure 6:**
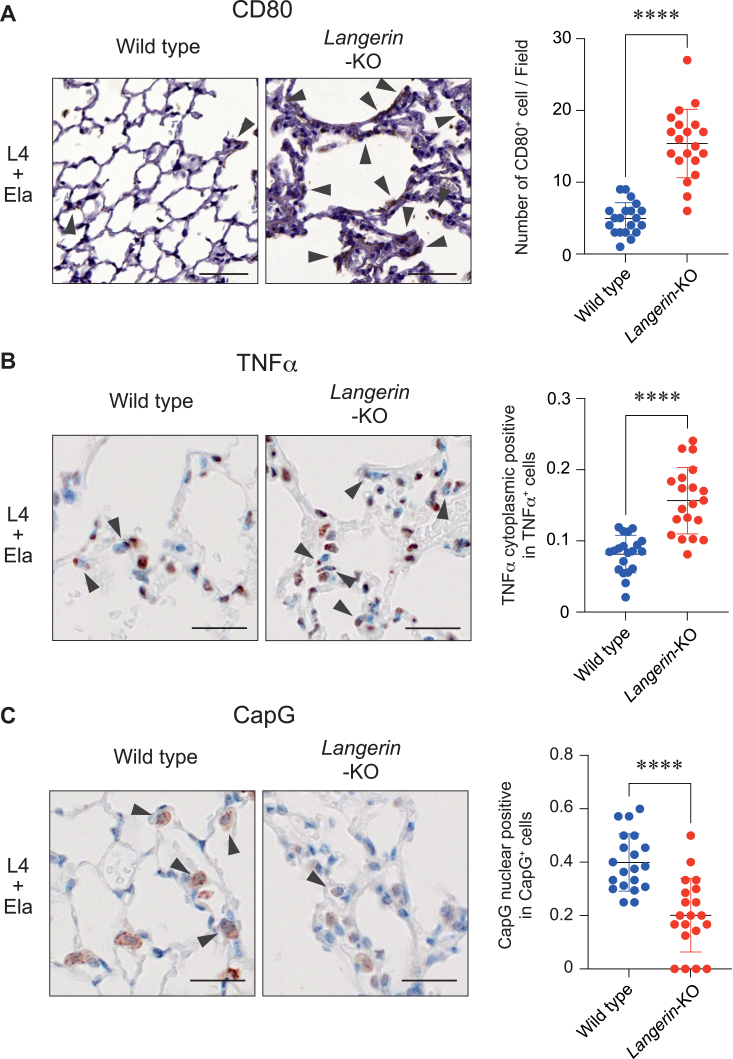
**L4 mitigates the inflamed state through the langerin-CapG axis in the lung of the emphysema mouse model.***A*, representative images of the staining for CD80 in the lung sections of wild type and *langerin*-KO mouse after administration of L4 and elastase. Arrowheads indicate CD80-positive cells. The number of CD80-positive cells were counted in random 20 fields in each mouse. ∗∗∗∗*p* < 0.0001. Bars indicate 50 μm. *B*, representative images of the staining for TNFα in the lung sections of wild type and *langerin*-KO mouse after administration of L4 and elastase. The number of cells expressing TNFα in cytoplasmic region were counted in 20 randomly selected fields in each mouse and the ratio against the total cell number was shown. ∗∗∗∗*p* < 0.0001. Bars indicate 20 μm. *C*, representative images of the staining for CapG in the lung sections of wild type and *langerin*-KO mouse after administration of L4 and elastase. The number of cells expressing CapG in nuclear were counted in 20 randomly selected fields in each mouse and the ratio against total CapG positive cells was shown. ∗∗∗∗*p* < 0.0001. Bars indicate 20 μm.

**Figure 7:**
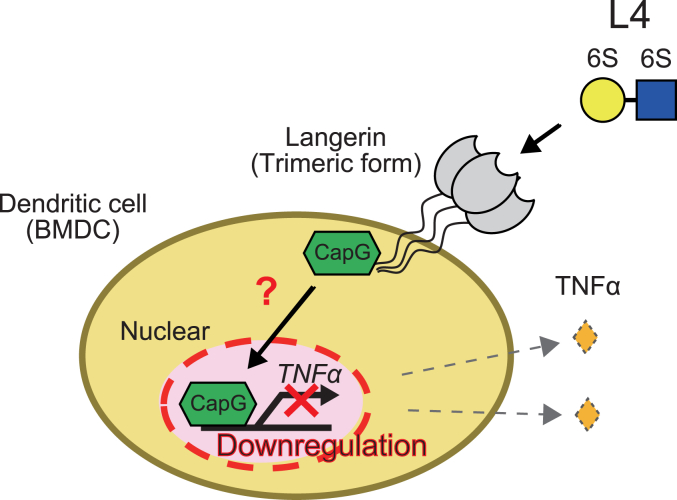
**Graphical summary of this study.** In dendritic cells (BMDCs), L4 promotes the nuclear localization of CapG through langerin. In the nucleus, CapG functions as an inhibitory transcription factor for *TNFα* gene. The L4-langerin-CapG axis results in the downregulation of TNFα, which contributes to the suppression of the inflammatory state of the lung and mitigation of symptoms associated with emphysema.

In BMDCs, L4 suppressed the gene expression of *TNFα* through the langerin-CapG axis, as demonstrated above (Figs. 3 and 4). We hypothesized that the downregulation mechanism for *TNFα* by L4 would also function in the emphysema mouse model. To address this, we analyzed the inflammatory status and the dynamic state of CapG in the lungs in the emphysema mouse model. CD80 is often used as a marker for the activation of antigen-presenting cells including dendritic cells (24). As shown in Figure 6*A*, we stained the lung sections of *langerin*-knockout mice and wild type mice with an anti-CD80 antibody after the administration of L4 and elastase. Interestingly, the number of CD80 positive cells was dramatically increased in the lungs of *langerin*-knockout mice compared to that of wild type mice. We also analyzed the expression of TNFα by immunohistochemical staining of lung sections and found that the lungs of *langerin*-knockout mice expressed TNFα more strongly than the wild type mice (Fig. 6*B*). Moreover, in the analysis of the subcellular localization of CapG in the lung, reduced nuclear expression of CapG was observed in the *langerin*-knockout mice compared to that in wild type mice (Fig. 6*C*). These data demonstrate the anti-inflammatory function of L4 through the langerin-CapG axis in *in vivo* and strongly suggest that the langerin-CapG axis represents a therapeutic target for emphysema (Fig. 7).

## Discussion

In this study, CapG was identified as a molecule that cooperates with langerin. It was also demonstrated that L4 enhanced the function of CapG to suppress the *TNFα* gene in BMDCs. Notably, both human and murine langerin harbor a proline-rich domain (PRD) inside their cytoplasmic region. The PRD is generally known to be a unique and functional amino acid domain for docking with a multitude of signaling molecules (25, 26). To determine whether the PRD of langerin is involved in the binding with CapG, we again performed immunoprecipitation with an anti-langerin antibody after the overexpression of human CapG and the PRD-deleted mutant of human langerin (ΔPRD-langerin) in HEK293 cells. However, contrary to our expectations, we observed an association between CapG and ΔPRD-langerin in this analysis (Fig. S7), which indicates that PRD of langerin is not required for langerin to bind with CapG. CapG was originally identified as playing a role in the organization of actin filament in macrophages (27), which suggests that CapG mediates the internalization and recruitment of langerin to lysosomes. Also, CapG could be phosphorylated by PKC (protein kinase C) (28). The dynamics of now CapG is regulated by phosphorylation has not been uncovered, although the phosphorylation would be one of the molecular mechanisms responsible for changing the subcellular localization of CapG. The possibility that phosphorylation of CapG is involved in the transport into the nuclear and function for translational regulation cannot be excluded, but further experiments would be needed to confirm this possibility. Notably, Huang S. and colleagues reported that CapG functions as a transcription factor in breast cancer (29). Upregulation of the *STC-1* (*stanniocalcin-1*) gene by CapG in cancer cells enhances breast cancer metastasis. In this report, our ChIP-qPCR data (Fig. 3*A*), and that the *CAPG* gene potentially contains a homology sequence with basis helix-loop-helix family DNA-binding proteins (bHLH) (30) constitute strong pieces of evidence to suggest that CapG plays a role as a transcription factor in immune cells and cancer cells.

In patients with emphysema and/or COPD, a subset of dendritic cells expressing langerin selectively accumulate in the small airways (31). This langerin-expressing subset uniquely expresses IgE receptors, which might be involved in the cross-presentation of antigens to CD8^+^ T cells (32). Other lectin receptors in emphysema and/or COPD have also been studied. The production of sialic acid-binding immunoglobulin-type lectin-9 (Siglec-9) is also increased in alveolar neutrophils in the lungs of COPD patients. Inflammatory cytokines, for example, IL-6, IL-8, and TNFα induce the expression of Siglec-9 and its soluble form in neutrophils, which contributes to the negative feedback loop by suppressing neutrophil activation (33). C-type lectin domain family five-member A (CLEC5A) is also involved in the development of COPD. Using *Clec5a*-deficient mice, it was demonstrated that Clec5a was required for the expression of inflammatory cytokines that are linked to the enlargement of airspaces in the lungs of a COPD model (34). An increase in the levels of these lectin receptors, especially langerin, in the lung of patients with emphysema and/or COPD would be expected to mitigate the inflammatory state of the lung. However, there have not been any constructive research that clearly demonstrates the critical function of lectin receptors against emphysema and COPD.

Tobacco smoke can strongly induce the *TNFα* gene, which is involved in the onset and progression of emphysema and COPD (35, 36). In the lung, a variety of immune cells including macrophages, monocytes, and dendritic cells primarily produce TNFα as well as other types of cells, for example, alveolar epithelial cells also have the potential to express TNFα. Otherwise, the bronchioles mainly express the receptors for TNFα (37). In the analysis using a transgenic mouse that expresses *TNFα* in alveolar type II cells under the control of the human surfactant protein C promoter, the overexpression of *TNFα* resulted in the development of pulmonary emphysema by the activation of the elastase (38). In addition, it was reported that an increased expression of *TNFα* contributed to the progressive disease state in COPD patients (39). For these studies, the blockade of TNFα is considered to be a therapeutic strategy against emphysema and COPD (40). In this study, we demonstrated that L4 suppressed the expression of *TNFα* through a langerin-CapG axis in dendritic cells. This is strong evidence to suggest that L4 is a potential candidate as a novel TNFα suppressor. In the clinical setting, corticosteroids are often used to modulate the inflammatory state of emphysema and COPD by the suppression of *TNFα*. The treatment with corticosteroids inhibits the accumulation and activation of neutrophils and macrophages in the lung of these patients. Intriguingly, in a previous study, we reported that L4 also inhibited the accumulation of neutrophils and macrophages in the bronchoalveolar lavage fluid of an elastase-induced emphysema model mouse. The level of this inhibitory effect of L4 was similar to that of the corticosteroid, dexamethasone (17). In this study, we also demonstrated that the molecular mechanism of L4 involves the suppression of inflammation through langerin. As also reported in this study, uncovering the molecular mechanism of the target should be very important in terms of understanding the biological processes involved and in estimating the undesirable effects associated with its administration.

Glycomimetic is a term indicating a type of scientific innovation in which glycan analogs are used to improve biological functions (41). Some types of glycans that target C-type lectin receptors are being developed as novel specific delivery systems for this purpose (42, 43). Wamhoff and co-workers identified heparin-derived monosaccharides as glycomimetic langerin ligands and reported that it is possible to deliver liposomes modified with its glycomimetic ligand specifically to Langerhans cells (44, 45). As described above, L4 is also a glycan analog that mimics the glycosaminoglycan of keratan sulfate proteoglycan. Keratan sulfate is normally expressed in the human body, especially in joints, corneas, and brain. Hence, the administration of L4 would not be expected to show any severe side effects (46). L4 would be stable because there is no naturally produced enzyme to degrade it, and additionally, the molecular weight of L4 is small, therefore making tissue permeability of L4 high. These properties would be strong advantages for the development of L4-based glycomimetics as anti-inflammatory drugs. There are many inflammatory diseases that are exacerbated by the over-activation of dendritic cells in addition to emphysema. Also, it is known that chronic inflammation in the tissues increases the risk of cancer. Considering these issues, we conclude that L4 would be an ideal candidate for use in drug discovery. Our research group plans to continue our research into L4 for subjects who are suffering from abnormal inflammation including emphysema and COPD.

## Experimental prodecures

### Cells and cell culture

Human promyelocytic leukemia cell line: HL-60, human monocytic leukemia cell line: THP-1, and human embryonic kidney cell line: HEK293 were obtained from the RIKEN BioResource Research Center (Tsukuba, Japan). Human diffuse histiocytic lymphoma cell line: U937 was obtained from JCRB Cell Bank. HL-60, THP-1, and U937 cells were cultured in RPMI-1640 (FUJIFILM Wako Pure Chemical Corporation) supplemented with 10 % (v/v) fetal bovine serum (FBS) and 1 % (v/v) penicillin-streptomycin (Thermo Fisher Scientific) at 37 °C under 5 % CO_2_. HEK293 cells were cultured in DMEM (FUJIFILM Wako) supplemented with 10 % (v/v) of fetal bovine serum (FBS) and 1 % (v/v) penicillin-streptomycin.

### Isolation and differentiation of bone marrow cells

Bone marrow cells were isolated from 10 to 20 weeks old male mice (C57BL/6J). The isolated cells were cultured in RPMI-1640 (FUJIFILM Wako) supplemented with 10 % (v/v) fetal bovine serum (FBS), 1 % (v/v) penicillin-streptomycin (Thermo Fisher Scientific), 50 μM 2-mercaptoethanol (FUJIFILM Wako), and a 20 ng/ml suspension of mouse granulocyte macrophage colony-stimulating factor (Abcam). The culture medium was replaced every 3 days. After incubation for totally 8 days, floating cells were analyzed as BMDCs (bone marrow-derived dendritic cells).

### Flow cytometry

BMDCs were washed twice with ice-cold PBS and incubated with an anti-CD16/CD32 antibody (Thermo Fisher Scientific, catalog number 14–0161–85) to block the non-specific binding of antibodies in further procedures. After washing with ice-cold PBS, the cells were incubated with an anti-langerin antibody labeled with phycoerythrin (BioLegend Inc, catalog number 144203) and an anti-CD11c antibody labeled with allophycocyanin (Thermo Fisher Scientific, catalog number 17–0114–82) for 1 h on ice. After washing twice with ice-cold PBS, cells were resuspended in 500 μl of ice-cold PBS and analyzed with BD FACSCalibur (BD Biosciences).

### Quantitative reverse transcription PCR

RNAs were extracted from cells by using RNeasy Mini Kit (QIAGEN, Venlo, Netherlands). Reverse transcription was performed with oligo dT primer and SuperScript IV Reverse Transcriptase (Thermo Fisher Scientific) following manufacturer’s protocol. Real-time PCR analysis was also carried out with THUNDERBIRD Next SYBR qPCR Mix (TOYOBO) and analyzed with 7500 Real-Time PCR System (Applied Biosystems). Primers for mouse *IL-6* were 5′-CTCATTCTGCTCTGGAGCCC-3′ and 5′-TGCCATTGCACAACTCTTTTCT-3′, for mouse *TNFα* were 5′-GTAGCCCACGTCGTAGCAAA-3′ and 5′-TAGCAAATCGGCTGACGGTG-3′, for mouse *IL-10* were 5′-GGCGCTGTCATCGATTTCTC-3′ and 5′-CGGAGAGAGGTACAAACGAGG-3′, for human *IL-6* were 5′- CCTTCGGTCCAGTTGCCTT-3′ and 5′- GTTGTTTTCTGCCAGTGCCT-3′, for human *TNFα* were 5′- CCTCTCTCCCCTGGAAAGGA-3′ and 5′- CTACAGGCTTGTCACTCGGG-3′, for human *IL-10* were 5′- CAAAAGAAGGCATGCACAGCTC-3′ and 5′- ACTGGATCATCTCAGACAAGGC-3′, for mouse *Mcp-1* were 5′- CACTCACCTGCTGCTACTCA-3′ and 5′- AGACCTTAGGGCAGATGCAG-3′, for mouse *IL-1α* were 5′-CCCGTGTTGCTGAAGGAGTTG-3′ and 5′-TGACTCAGGGTGAGGAAGGTT-3′, for mouse *IL-1β* were 5′-TGCCACCTTTTGACAGTGATG-3′ and 5′-AAGGTCCACGGGAAAGACAC-3′, for mouse *IFN-β* were 5′-TGGGAGATGTCCTCAACTGC-3′ and 5′-ACTACCAGTCCCAGAGTCCG-3′.

### PCR for mouse genotyping

A portion of the murine tail was incubated with 200 μl of DNA extraction buffer (0.1 M Tris-HCl (pH 8.0), 1 mM EDTA (pH 8.0), 0.5 % (v/v) Tween20) and 100 μg of Proteinase K (FUJIFILM Wako) at 55 °C overnight. The tails were then heated at 95 °C for 10 min to inactivate Proteinase K. Using the resulting solution, PCR was performed with MightyAmp DNA Polymerase (Takara Bio Inc). Primers targeting *exon No.3* of *langerin* gene were 5′- GAGGGTGCGTTCTCAGATCC-3′ and 5′- TGCTCAAAACAGGTAGGGGC-3′, and targeting deleted allele of *langerin* gene were 5′- TGTGTGGTAGCCCCTAATGC-3′ and 5′-CCTGGAGACGTGAGCAGAAA-3′. PCR products were separated by agarose gel electrophoresis at 100 V for 20 min and visualized with Midori Green Direct (NIPPON Genetics Co, Ltd).

### Preparation of cell lysates and immunoprecipitation

Cells were washed 3 times with ice-cold PBS and lysed with cell lysis buffer (1% NP-40 in TBS) that included protease inhibitors (Roche, Basel, Switzerland) and PhosSTOP phosphatase inhibitor (Roche). The lysates were sonicated briefly and centrifuged at 15,000*g* at 4 °C for 5 min to remove the insoluble materials. The protein concentration of the lysate was determined with a BCA protein assay kit (BioRad Laboratories). For immunoprecipitation, a part of lysate was incubated with an anti-langerin antibody (BioLegend Inc, catalog number 144202) or non-relevant isotype IgG (Santa Cruz Biotechnology, Inc) at 4 °C overnight under rotation. Next day, 20 to 50 μl of Protein G Sepharose four Fast Flow (GE Healthcare) was added into the lysate and incubated for more 2 h at 4 °C under rotation. After washing 3 times with cell lysis buffer, Protein G Sepharose was added to the sample buffer (50 mM Tris (pH6.8), 2 % (w/v) SDS, 2.5 % (v/v) 2-mercaptoethanol, 9 % (v/v) glycerol) and heated at 95 °C for 5 min.

### Preparation of cytoplasmic and nuclear fractions

Cells were washed 3 times with ice-cold PBS. After washing, 500 μl of hypotonic buffer (20 mM Tris-HCl (pH 7.4), 10 mM NaCl, 3 mM MgCl_2_) including protease inhibitors (Roche) was added and then incubated on ice for 15 min. Twenty-5 μl of 10 % NP-40 was added and the resulting suspension was vortexed briefly. After incubation on ice for more 3 min, the resulting solution was centrifuged at 3000 rpm (TOMY TMP-1 rotor) at 4 °C for 10 min. The supernatant was transferred into new tube as cytoplasmic fraction. Additionally, 100 μl of RIPA buffer (50 mM Tris-HCl (pH 8.0), 150 mM sodium chloride, 0.5 % (w/v) sodium deoxycholate, 0.1 % (w/v) sodium dodecyl sulfate, 1 % (v/v) NP-40) including protease inhibitor (Roche) was added into the pellet. After incubation on ice for 30 min, the resulting solution was centrifuged at 14,000*g* at 4 °C for 30 min. The supernatant was transferred into new tube as nuclear fraction.

### Mass spectrometry

The immunoprecipitates prepared from BMDCs with an anti-langerin antibody were separated by SDS-PAGE using 10 % (w/v) acrylamide gels. Silver staining was then performed with Pierce Silver Stain for Mass Spectrometry (Thermo Fisher Scientific) following manufacturer’s protocol. The bands were cut out and destaining was also performed by using regents in the Pierce Silver Stain for Mass Spectrometry. The resulting piece of the gel was taken to dryness with a KPI Rotary Vacuum Miro-concentrator RMC-24 (KOIKE PRECISION INSTRUMENTS) and EYELA Cold Trap UT-1000 (TOKYO RIKAKIKAI CO LTD). Proteins in the gel pieces were digested with trypsin for Mass Spectrometry (Thermo Fisher Scientific) after reduction with dithiothreitol and alkylation with iodoacetamide. The tryptic peptides were trapped using a short ODS column (PepMap 100; 5 μm C18, 5 mm × 300 μm ID; Thermo Fisher Scientific) and then separated using an ODS column (Nano HPLC Capillary Column; 3 μm C18, 120 mm × 75 μm ID; Nikkyo Technos) using nano-liquid chromatography (nanoLC) (Ultimate 3000; Thermo Fisher Scientific). The mobile phases for separation were solvent A (0.1% formic acid) and solvent B (0.1% formic acid in acetonitrile). After loading tryptic peptides to trap column with 0.1% TFA for 3 min at a flow rate of 30 μl/min, the concentrated tryptic peptides were eluted from trap column and separated on separation column using a sequence of isocratic and a linear gradient: 0 to 3 min, solvent A; 3 to 35 min, 0 to 35% (v/v) solvent B; and increasing to 90% solvent B for 10 min and re-equilibrated with solvent A for 15 min. The eluate from separation column was introduced continuously into a nanoESI source and analyzed by MS and MS/MS (LTQ Orbitrap XL; Thermo Fisher Scientific). MS and MS/MS spectra were obtained in the positive ion mode using Orbitrap (mass range: m/z 300–1500) and Iontrap (data dependent scan of top five peaks using CID), respectively. The voltage of the capillary source was set at 1.6 kV, and the temperature of the transfer capillary maintained at 200 °C. The capillary voltage and tube lens voltage were set at 30 V and 80 V, respectively. Assignment of MS/MS data to tryptic peptides was performed by Proteome Discoverer 2.4 (Thermo Fisher Scientific) using Mascot search engine 2.5.1 (Matrix Science) on UniProt data base. Doubly and triply charged peptide ions were subjected to the database search with precursor and fragment ion mass tolerance of ±10 ppm and ±0.8 Da, respectively, with static modification (carbamidomethylation of cysteine) and dynamic modification (oxidation of methionine and deamidation of asparagine and glutamine). The significance threshold on Proteome Discoverer for Mascot search was set at *p* < 0.05 and one and two missed trypsin cleavage was allowed.

### Western blotting

Cell lysates were separated by SDS-PAGE using 10 % (w/v) acrylamide gels. After transferring to a nitrocellulose membrane (Merck Millipore, Burlington, MA), the membrane was blocked with 2 % (w/v) bovine serum albumin (Nakalai tesque, Inc) or 5 % (w/v) skim milk (Nakalai tesque, Inc) in TBST (0.1 % (v/v) Tween20 in TBS). The membrane was then incubated with an anti-CapG antibody (GeneTex, Irvine, CA, catalog number GTX114301), an anti-Lamin B1 antibody (BioLegend Inc, catalog number 8969801), or an anti-GAPDH antibody (Merck Millipore, catalog number MAB374). After washing 4 times with TBST, the membrane was then incubated with an anti-rabbit or -mouse IgG labeled with horseradish peroxidase (GE Healthcare). To visualize the bands, the membrane was analyzed with Amersham ECL Prime Western Blotting Detection Reagents (GE Healthcare) and ChemiDoc MP Imaging System (BioRad Laboratories).

### Immunocytochemistry

Cells were washed 3 times with ice-cold PBS and fixed with 4 % (w/v) paraformaldehyde in PBS. After washing 3 times with PBS, cells were incubated for 10 min with 0.1 % (v/v) TritonX-100 in PBS at room temperature, The resulting cells were then washed with PBS and blocked with 2.5 % w/v bovine serum albumin (Nakalai tesque, Inc) in PBS. After blocking, cells were incubated with an anti-CapG antibody (Santa Cruz Biotechnology, Inc, catalog number sc-166428) and an anti-mouse IgG labeled with Alexa 594 (Thermo Fisher Scientific, catalog number A21044). To stain the nucleus, cells were also incubated with DAPI (DOJINDO LABORATORIES). The stained cells were analyzed with confocal microscopy, FV10i (Olympus Corporation) or LSM 900 (ZEISS).

### Chromatin immunoprecipitation-quantitative PCR (ChIP-qPCR)

Cells were washed twice with PBS and fixed with 1 % (v/v) formaldehyde in PBS. After incubation at room temperature for 10 min, quenching was performed by incubation with 0.125 M glycine in PBS. The cells were then lysed with RIPA buffer including protease inhibitors (Roche) and the DNAs were fragmented by sonication. The size of fragmented DNAs was confirmed as approximately 600 to 1000 bp. The resulting solution was centrifuged at 15,000 g at 4 °C for 10 min to remove debris. The protein concentration was measured by using BCA protein assay kit (BioRad Laboratories) and a part of lysate including 2 mg of total protein was incubated with 4 μg of anti-CapG antibody (Santa Cruz Biotechnology, Inc, catalog number sc-166428) or non-relevant isotype IgG (Santa Cruz Biotechnology, Inc) overnight at 4 °C under rotation. On the next day, 30 μl of Protein G Sepharose four Fast Flow (GE Healthcare) was added and the resulting solution incubated at 4 °C for more 2 h under rotation. The resulting solution was centrifuged at 4 °C for 30 s and the supernatant was removed. Five hundred μl of TE was added into the Protein G Sepharose, then, RNaseA (50 μg/ml) (Merck Sigma-Aldrich, St Louis, MO) treatment at 37 °C for 30 min and proteinase K (1.68 unit/ml) (FUJIFILM Wako) treatment at 55 °C for 1 h were carried out. After heating at 95 °C for 10 min for inactivation proteinase K, DNA fragments were purified and concentrated by using phenol-chloroform. The pellet of DNA was resuspended in 50 μl of TE and a 2 μl aliquot was analyzed by qPCR. Primers targeting the promoter region of mouse *IL-6* gene were 5′-TGCTCATGCTTCTTAGGGCT-3′ and 5′-TGGGGCTGATTGGAAACCTT-3′, targeting the transcription factor binding site of mouse *IL-6* gene were 5′-GACTGAGCCTAAGGGTGCAT-3′ and 5′-ACCACTAGAGGGCCAAGTCA-3′, targeting promoter region of mouse *TNFα* gene were 5′-ACTGATGAGAGGGAGGCCAT-3′ and 5′-AACTGTAAGCGGGGCAATCA-3′, targeting promoter with the flank region of mouse *TNFα* gene were 5′-TGAGTCCTTGATGGTGGTGC-3′ and 5′-AGGGGATTATGGCTCAGGGT-3′, and targeting enhancer region of mouse *Mcp-1* gene were 5′- GGACTCAGCCGACTTACTGG-3′ and 5′- AAGGAAGTGGCCAAGGAACC-3′. The genome regions were identified by using the Ensembl genome browser.

### Generation of CapG knockout THP-1 cells

THP-1 cells were transfected with a pSpCas9(BB)-2A-GFP vector (Addgene, plasmid code 48138) harboring target sequences against the human *CAPG* gene by using jetPRIME (PolyPlus-transfection, Illkirch, France). After culturing for 2 days, GFP positive cells were enriched with a S3 Cell Sorter (BioRad Laboratories). Clonal cells then grown from single cells were prepared by limiting the dilution. Target sequences were 5′-GATCTGAAGACAGCATGTAC-3′ and 5′-GTGCCGTGCTGGCTGTGCAC-3′.

### Generation of langerin-knockout mice

The pX330-U6-Chimeric_BB-CBh-hSpCas9 vector (Addgene, plasmid code 42230) harboring target sequences against mouse *langerin* gene was implanted by micro-injected into embryonic stem cells. In the newborn mice, the mutation of *langerin* gene was confirmed by PCR and DNA sequencing analysis. Dr Satoru Takahashi (Tsukuba University) and Dr Yasunori Chiba (AIST: National Institute of Advanced Industrial Science and Technology) kindly contributed for generation of *langerin*-knockout mice. The genetic background of mice is C57BL/6J.

### Intratracheal administration and computed tomography analysis

Under general anesthesia, a 100-μl solution of L4 in saline (10 mg/ml) and 100 μl of elastase from the porcine pancreas in saline (40 U/ml) (FUJIFILM Wako) were administrated into the tracheae by spray (17). After 21 days, CT analyses were performed with ALOKA Latheta Laboratory CT LCT-200 (Hitachi, Ltd). The area indicated by the intensity value between −900 to −610 was calculated as low attenuation area. The male mice (10–20 weeks old) were analyzed.

### Preparation of paraffin sections and immunohistochemistry

Mice were euthanized by inhaling carbon dioxide gas. For the fixation of lung tissues, 10 % (v/v) formalin natural buffer solution (FUJIFILM Wako) was injected into the lung at a constant rate through the respiratory tract. After removing the lung tissues, paraffin blocks were prepared using an automatic fixation and embedding machine RH-12 (Sakura Finetek Japan Co, Ltd) and HistoCore Arcadia H (Leica Biosystems). Four μm-thick paraffin sections were prepared with Microm HM 360 Automated Microtome (Marshall Scientific LLC.). For immunohistochemistry, the sections were microwaved for epitope retrieval and deparaffinization was then performed. After washing with water, the sections were saturated in an Antigen Unmasking Solution (Vector Laboratories) and microwaved again. The sections were then incubated with 1 % (v/v) H_2_O_2_ in MeOH for 5 min at room temperature. After washing with PBST (0.05 % (v/v) Tween 20 in PBS), the sections were blocked with 1.5 % (w/v) bovine serum albumin (Nacalai tesque) in PBST. The resulting sections were incubated with an anti-CD80 antibody (Santa Cruz Biotechnology, Inc, catalog number sc-376012), an anti-TNFα antibody (Novus Biologicals, Littleton, CO, catalog number NBP1-19532SS), or an anti-CapG antibody (Santa Cruz Biotechnology, Inc, catalog number sc-166428). After incubation at 4 °C overnight, the sections were washed 4 times with PBST and incubated with Dako EnVision + Dual Link System-HRP (Agilent Technologies, Inc) at room temperature for 30 min. After washing with PBST, the sections were visualized by means of a Liquid DAB+ Substrate Chromogen System (Agilent Technologies, Inc). Counter staining with Mayer’s hematoxylin solution (ScyTec Laboratories, Inc) was also performed. The sections were then dehydrated and encapsulated with Multi Mount 220 (Matsunami Glass Ind., Ltd) and the resulting sections were observed by microscopy, BZ-X710 (KEYENCE CORPORATION).

### Statistical analysis

All data are presented as the mean ± standard deviation. For comparison between multiple groups, the one-way ANOVA test was performed. For comparison between two groups, the student’s *t* test analysis was performed.

### Study approval

All studies using mice were approved by the local committee at Osaka International Cancer Institute (Approval number, 22,030,316).

## Data availability

All data are contained with the manuscript or associated supplementary files.

## Supporting information

This article contains supporting information.

## Conflicts of interest

The authors declare no conflicts of interest associated with this study.
