# Supplementary material for: Involvement of langerin in the protective function of a keratan sulfate-based disaccharide in an emphysema mouse model
Source: J Biol Chem. 2023 Jul 15;299(8):105052. doi: 10.1016/j.jbc.2023.105052 (PMC10448169; doi:10.1016/j.jbc.2023.105052)
Supplement: Supplementary information [file mmc1.docx]

**Supplementary information**

**Supplementary Figures**

**Supplementary Figure S1. *Structure of the glycosaminoglycan of keratan sulfate proteoglycan and L4.***

Keratan sulfate is classified into three types, corneal KS-I, skeletal KS-II, and brain KS-III. The terminal glycan structure is unknown in KS-III. L4: galactose-6-sulfate β1,4-linked to *N*-acetylglucosamine-6-sulfate, is a component of glycosaminoglycan of keratan sulfate proteoglycan.

**Supplementary Figure S2. *CapG was determined as a langerin-binding molecule in HEK293 cells.***

Western blotting of the immunoprecipitates prepared from HEK293 cells. Cells were overexpressed with both mouse langerin and mouse CapG and immunoprecipitation was then performed with an anti-langerin antibody. The immunoprecipitates were blotted with an anti-CapG antibody. Arrow indicates CapG.

**Supplementary Figure S3. *CapG is associated with langerin in THP-1 cells.***

**(A)** Western blotting of the immunoprecipitates with an anti-CapG antibody. THP-1 cells were lysed and immunoprecipitation was performed with an anti-langerin antibody or non-relevant IgG. Arrow indicates CapG. **(B)** Representative confocal microscopic images of THP-1 cells. Immunocytochemical staining was performed with an anti-CapG antibody (Red) and an anti-langerin antibody (Green). Counterstaining with DAPI was also performed. To decrease non-specific intracellular signals, the permeabilization step was skipped after the fixation. Bar indicates 10 µm.

**Supplementary Figure S4. *Primer design for ChIP-qPCR analysis in Figure 3A.***

**Supplementary Figure S5. *Genomic DNA sequence of langerin gene in langerin-knockout mice.***

Genomic DNA sequence of *langerin* gene (Chromosome 6: 83,653,091 – 83,652,575 and 83,651,330 – 83,650,794, revers strand) in wild type and langerin-knockout mice. Genomic DNAs were extracted from the tails and a DNA sequencing analysis performed. Target sequences of Cas9 for genome editing were indicated with squares. Yellow is exon No.3 and purple is exon No.4 of *langerin* gene. DNA sequence indicated with dushed line was deleted in *langerin*-knockout mice.

**Supplementary Figure S6. *L4 suppresses the pulmonary fibrosis in an emphysema mouse model.***

Representative images of Masson’s trichrome staining. The lung sections prepared from wild type and *langerin*-KO mice after the administration of L4 and staining for fibrosis was carried out. Area of fibrosis was visualized as blue. Bars indicate 50 µm.

**Supplementary Figure S7. *Proline-rich domain (PRD) of langerin is not involved in the binding with CapG.***

Western blotting of the immunoprecipitates prepared from HEK293 cells examined with an anti-CapG antibody. Cells were overexpressed with mouse CapG and mouse langerin or PRD-deleted mutant of murine langerin (ΔPRD-langerin), then, immunoprecipitation was performed with an anti-langerin antibody. Arrow indicates CapG.

**Method for DNA sequencing analysis**

A portion of a tail was incubated with 200 µl of DNA extraction buffer for tail (0.1 M Tris-HCl (pH 8.0), 1 mM EDTA (pH 8.0), 0.5 % Tween20) and 100 µg of Proteinase K (FUJIFILM Wako Pure Chemical Corporation, Osaka, Japan) at 55 ℃ overnight. The resulting tissue was then heated at 95 ℃ for 10 min to inactivate Proteinase K. DNA fragment of *langerin* gene was amplificated by PCR using MightyAmp™ DNA Polymerase (Takara Bio Inc., Shiga, Japan). Primers were 5’-TGTGTGGTAGCCCCTAATGC-3’ and 5’-CCTGGAGACGTGAGCAGAAA-3’. After purifying PCR products by agarose gel electrophoresis and by using QIAquick Gel Extraction Kit (QIAGEN, Venlo, Netherlands). DNA sequencing analysis was carried out with the same primers in PCR.

**Method for Masson’s trichrome staining**

Deparaffinization was firstly performed following the standard protocol. The lung sections were washed with water and incubated with the first mordant (MUTO PURE CHEMICAL CO., LTD., Tokyo, Japan) for 20 min. After washing with water, the sections were incubated for 10 min with Weigert’s iron hematoxylin solution (MUTO PURE CHEMICAL CO., LTD.). After washing with water, the sections were treated with 0.5% hydrochloric acid in 70% ethanol and washed again with water. The sections were then incubated for 30 sec with the second mordant. After washing with water again, the sections were incubated with 0.75% Orange G solution (MUTO PURE CHEMICAL CO., LTD.) for 1 min. After washing twice with 1% acetic acid, the sections were incubated with Masson’s stain solution B (MUTO PURE CHEMICAL CO., LTD.) for 20 min. After washing twice with 1% acetic acid again, the sections were incubated with 2.5% phosphotungstic acid solution (MUTO PURE CHEMICAL CO., LTD.) for 15 min. The sections were then washed twice with 1% acetic acid again and incubated with aniline blue solution (MUTO PURE CHEMICAL CO., LTD.) for 30 min. The sections were washed with 1% acetic acid twice and water once, dehydration was performed with ethanol following the standard protocol. The resulting sections were treated with xylene and encapsulated with Multi Mount 220 (Matsunami Glass Ind., Ltd., Osaka, Japan). The stained sections were observed by microscopy, BZ-X710 (KEYENCE CORPORATION).
